# Digitizing the Blue Light-Activated T7 RNA Polymerase System with a *tet*-Controlled Riboregulator

**DOI:** 10.1021/acssynbio.5c00142

**Published:** 2025-05-19

**Authors:** Sara Baldanta, Guillermo Rodrigo

**Affiliations:** † Institute for Integrative Systems Biology (I2SysBio), CSIC − University of Valencia, 46980 Paterna, Spain

**Keywords:** Antibiotic resistance, Optogenetics, Small RNA, Synthetic biology

## Abstract

Optogenetic systems offer precise control over gene expression, but leaky activity in the dark limits their dynamic range and, consequently, their applicability. Here, we enhanced an optogenetic system based on a split T7 RNA polymerase fused to blue-light-inducible Magnets by incorporating a *tet*-controlled riboregulatory module. This module exploits the photosensitivity of anhydrotetracycline and the designability of synthetic small RNAs to digitize light-controlled gene expression, implementing a repressive action over the translation of a polymerase fragment gene that is relieved with blue light. Our engineered system exhibited 13-fold improvement in dynamic range upon blue light exposure, which even raised to 23-fold improvement when using cells preadapted to chemical induction. As a functional demonstration, we implemented light-controlled antibiotic resistance in bacteria. Such integration of regulatory layers represents a suitable strategy for engineering better circuits for light-based biotechnological applications.

## Graphical abstract

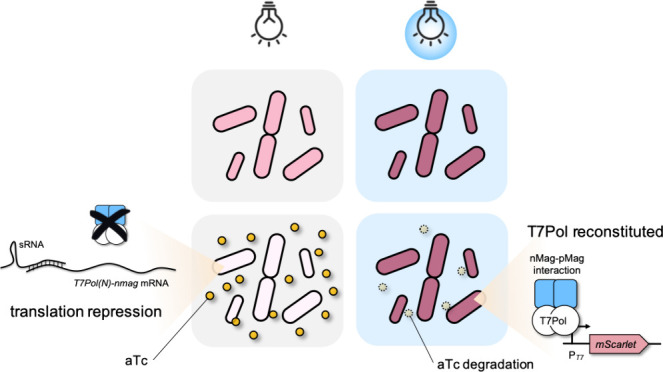

## Introduction

Engineered optogenetic systems represent a significant biotechnological advance to control gene expression and protein activity *in vivo* with high spatiotemporal precision.^1^ In contrast to chemical inducers, light is not limited by diffusion and shows higher orthogonality in addition to being cost-effective. Optogenetic systems broadly rely on light-controlled protein–protein interactions (including dimerization),^2,3^ RNA–protein interactions,^4^ protein modifications (*e.g*., phosphorylation),^5^ enzymatic activity (through conformational changes),^6^ and ion channel transport.^7^ Across different organisms, these mechanisms have been employed to regulate gene expression by reconstituting active transcription factors,^8,9^ with applications in metabolic engineering,^10,11^ to promote physical cell–cell interactions through membrane proteins,^12,13^ to edit the genome by reconstituting the Cre recombinase or Cas9 nuclease,^14,15^ and even to induce macromolecular phase separation for chromatin reconfiguration.^16^ However, a major challenge faced by these systems is the leaky activity in the absence of light, which often results in a limited dynamic range that precludes a wide applicability. This seems particularly problematic when unintended enzyme expression imposes toxicity due to the high metabolic burden or off-target effects. Overcoming this limitation requires optimization of light-responsive elements and integration of additional regulatory layers.

One relevant light-inducible transcriptional program in bacteria is the Opto-T7 system.^8^ It consists of a split T7 RNA polymerase (T7Pol) coupled to the light-inducible Magnets, which are engineered protein domains (nMag and pMag) that heterodimerize upon blue light stimulation.^2^ An alternative version of the system was implemented with the native homodimerizing photoreceptor Vivid from the filamentous fungus *Neurospora crassa*.^17^ The relevance of the Opto-T7 system lies in the versatility of using T7Pol to express any gene, a relatively high dynamic range of the response, and reduced cell–cell variability. Of note, the Opto-T7 system has also been implemented in mammalian cells.^18^ However, despite extensive optimization of Magnets and adjustment of protein domain production with different *cis*-regulatory regions, significant output expression levels under dark conditions persist. Thus, it would be convenient to investigate the use of additional regulatory elements to try to improve the Opto-T7 system.

In this work, we exploited the photosensitivity of anhydrotetracycline (aTc) to engineer a *tet*-controlled riboregulatory module that allows minimization of the activity of the Opto-T7 system under dark conditions. On the one hand, dynamic and modular optogenetic control circuits have been developed owing to the ability of ultraviolet light to fully degrade aTc, thereby reducing complexity by working with a one-component regulatory system.^19^ On the other hand, synthetic small RNA (sRNAs) are fully designable molecules^20^ that have been used to tightly control gene expression,^21,22^ allowing to work with highly toxic enzymes, and have been integrated with transcription factors to implement combinatorial regulation.^20,23^ All this capability has favored the development of sRNA-based genetic programs for metabolic rerouting^24^ and cell/virus-based therapeutic effector delivery.^25^ Importantly, we found that blue light is suitable to counteract intermediate aTc concentrations, which could still induce meaningful sRNA expression levels. We characterized the dynamic response of the new optogenetic system engineered here using *Escherichia coli* as a cell chassis. In addition, we demonstrated light-activated kanamycin resistance, while showing higher susceptibility in the dark than the original system.

## Results and Discussion

In the Opto-T7 system, two P_BAD_ promoters (excluding the catabolite activator protein binding site) are used to express the genes coding for the two protein moieties (*viz*., the N-terminus of T7Pol fused to nMag and pMag fused to the C-terminus of T7Pol; Figure 1A).^8^ The system is then activated by blue light and arabinose in a cell, producing the AraC transcription factor. Here, we designed an sRNA targeting the leader region of the gene encoding the N-terminus of T7Pol fused to nMag. In particular, the sRNA paired to the Shine–Dalgarno sequence and the start codon, thereby blocking ribosome binding (Figure S1). The whole sRNA molecule harbored a hammerhead ribozyme in the 5′ end for a self-cleavage process and a hairpin-formed transcription terminator able to recruit the Hfq RNA chaperone, as this strategy has been proven useful to obtain robust and efficient translation repression in bacteria.^26^ A P_Ltet_ promoter was used to express the sRNA, which can be tuned with aTc in a cell, producing the TetR transcription factor. Besides, we used the mScarlet red fluorescent protein as a reporter in our circuit, whose expression was driven by a T7 promoter. As a result, engineered bacterial cells growing in the presence of both arabinose and aTc under dark conditions would minimize the production of active T7Pol due to the post-transcriptional repression exerted by the sRNA. Upon blue light irradiation, aTc would be degraded, allowing TetR to repress the sRNA, and T7Pol would be reconstituted due to the heterodimerization of Magnets (Figure 1A).

**Figure 1:**
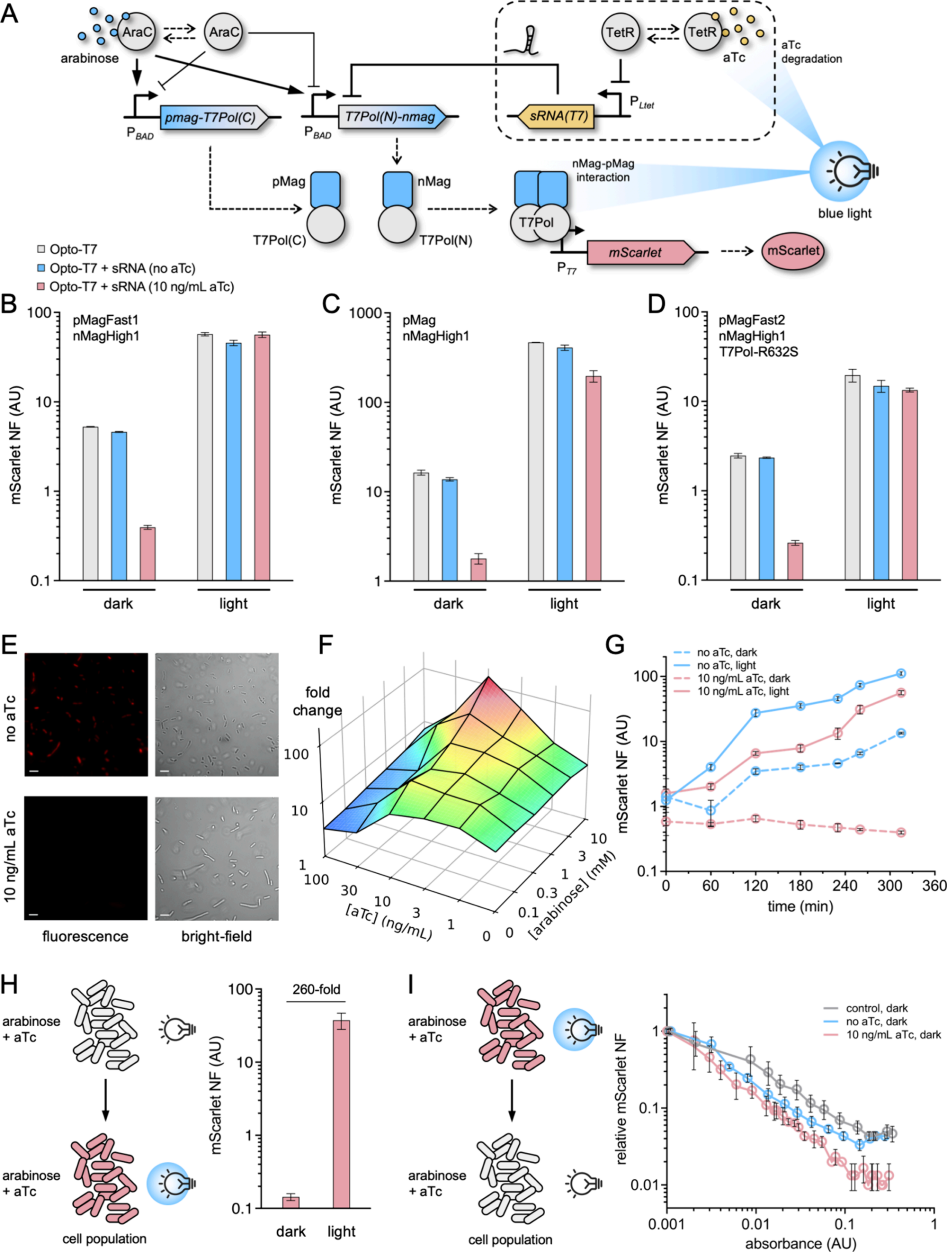
Digitized response of the Opto-T7 system through a *tet*-controlled riboregulator. A) Scheme of the engineered gene regulatory circuit in which blue light induces the heterodimerization of Magnets (pMag and nMag) to reconstitute T7Pol and the degradation of aTc to silence the *T7Pol*-targeting sRNA. B-D) Fluorescence-based reporter gene expression analyses (light/dark, with/without aTc). System implemented with pMagFast1 and nMagHigh1 in B), with pMag and nMagHigh1 in C), and with pMagFast2, nMagHigh1, and T7Pol-R632S in D). E) Microscopy images of single cells grown with and without aTc under dark conditions (fluorescence and bright-field images are shown). Scale bar, 5 μm. F) Surface plot of fold change as a function of the arabinose and aTc concentrations. G) Time-course responses of the system in different induction conditions. H) Fluorescence-based reporter gene expression analysis with preincubation with arabinose and aTc. I) Fluorescence-based analysis of induced reversibility with aTc. System implemented with pMagFast1 and nMagHigh1 in E–I). Represented data correspond to means ± standard deviations (*n* = 3). NF, normalized fluorescence. AU, arbitrary units.

We evaluated the dynamic range of the new system by measuring the change in red fluorescence of cells growing upon blue light irradiation (ON state) with respect to cells growing under dark conditions (OFF state) using a medium containing 10 mM arabinose and 10 ng/mL aTc (inducers added at time 0). There are different versions of the Opto-T7 system depending on the particular choice of sequences coding for Magnets and T7Pol.^2,27^ Using pMagFast1 and nMagHigh1, we obtained a 143-fold range, while the original system (without the *tet*-controlled riboregulatory module) displayed an only 11-fold range (Figure 1B). Of note, this was a 13-fold increase in performance. In particular, we observed that the output expression levels in the ON state remained roughly unchanged with and without sRNA, but in the OFF state the output expression was substantially reduced due to the repressive action of the sRNA (two-tailed Welch’s *t*-test, *P* < 10^–4^). This combination of Magnets has resulted fruitful to achieve low intrinsic association in the dark, adequate dissociation to ensure time-dependent switching with light, and high fold change of the response in steady state.^2^ Moreover, using pMag and nMagHigh1, we obtained a 111-fold range, while the original system only displayed a 28-fold range, leading to a 4-fold increase in performance (Figure 1C; assessment of sRNA action by two-tailed Welch’s *t*-test, *P* = 2·10^–4^). In this case, however, the output expression level in the ON state was slightly lower than without sRNA. Finally, with pMagFast2, nMagHigh1, and T7Pol-R632S (a mutation that maintains polymerase activity but reduces toxicity), we found a 6-fold increase in performance, going from an 8-fold range to a 51-fold range as a consequence of adding the riboregulatory module (Figure 1D; assessment of sRNA action by two-tailed Welch’s *t*-test, *P* < 10^–4^). With respect to pMagFast1, the use of pMagFast2 leads to a dissociation kinetics much faster between the two protein moieties, while the use of pMag to a dissociation kinetics much slower.^2^ This helps to explain the difference in absolute fluorescence observed in each case (see also Figure S2). One important consideration here is that the secondary light-canalizing route is irreversible as aTc is degraded in the process. Additional amounts of aTc should be added to the medium to recover a low output expression if light were switched off. Together, these results demonstrate that a *tet*-controlled riboregulator is a simple and powerful element to improve the functioning of optogenetic systems.

Focusing on the fold change, the system in which Opto-T7 is implemented with pMagFast1 and nMagHigh1 showed the best performance, so this system was used for further analysis. In applications requiring high expression levels, however, the system in which Opto-T7 is implemented with pMag and nMagHigh1 would be the right choice. Moreover, we noticed an impact of sRNA expression on cell growth (Figure S3). Yet, this was evidenced only when incubating under dark conditions in the presence of aTc. The chemical inducers by themselves or the expression of the native Opto-T7 system did not affect growth (Figure S4).

Next, we visualized the tight regulation of the *T7Pol*-targeting sRNA by microscopy imaging of single cells (Figure 1E). Cells were grown in the dark with and without aTc. We confirmed the low basal production of mScarlet when aTc was added. Some cells with elongated morphology were observed under this condition, which we attributed to the lower growth rate. We also characterized the system at a lower temperature (28 °C), finding again that the *tet*-controlled riboregulatory module led to a significant reduction of red fluorescence in the OFF state, going from a 4-fold range in the original system to a 10-fold range (Figure S5). Nonetheless, we observed more basal production of mScarlet in this case than at 37 °C, arguably due to a higher intrinsic association ability between the two Magnets at 28 °C. In this regard, subsequent developments might incorporate new mutations known to enhance the performance of Magnets as temperature varies.^28^ The functionality of the sRNA could also be affected due to a reduced ability for binding or ribozyme self-processing.

To further characterize the dynamic response of the engineered system, we determined the fold change of the response for a double concentration gradient of arabinose and aTc, leading to 36 combinations (Figure 1F; see also Figure S6). We found an optimal optogenetic response around 10 ng/mL aTc, indicating that this concentration is sufficient to produce a significant amount of sRNA for translation repression and adequate to be degraded by blue light in a short period of time. The global maximum was obtained at high levels of arabinose (10 mM). Indeed, the higher the arabinose concentration, the larger the dynamic range at intermediate aTc levels, stressing the efficacy of the sRNA to limit the production of the N-terminus of T7Pol in the OFF state. At 100 ng/mL aTc, blue light was not able to switch off the sRNA expression, obtaining only ∼5-fold values. To fully degrade such an amount of inducer, ultraviolet light would be required,^19^ at the cost of affecting cell physiology and integrity. In the absence of aTc, the fold change of the response with blue light remained nearly unchanged irrespective of the arabinose concentration (∼11-fold), despite the absolute red fluorescence values varied, thus indicating leaky association between Magnets in the dark. In addition, we monitored red fluorescence over time upon blue light irradiation (Figure 1G). In light conditions, fluorescence increase was slower when the medium contained aTc, as blue light requires time for a full degradation of the compound. Under dark conditions, fluorescence remained nearly constant at low levels over time when the sRNA was expressed, although a moderate increase was observed when the sRNA was repressed (because of the intrinsic ability of Magnets to dimerize). Thus, it turns out that the transcriptional activation by arabinose in conjunction with AraC is well compensated by the sRNA-based translation repression and that there is a suitable window of aTc concentrations that can derepress TetR-controlled transcription and be degraded by blue light in short times.

Moreover, a raise to 260-fold in dynamic range was obtained when a cell preincubation with the chemical inducers was applied (Figure 1H). In this latter case, the gene coding for the N-terminus of T7Pol was already silenced at the post-transcriptional level when stimulating with blue light. To demonstrate induced reversibility, cell cultures preincubated with blue light were diluted and regrown under dark conditions, showing how the addition of aTc to resume the sRNA expression led to lower fluorescence levels at the end (Figure 1I).

Motivated by these results, we constructed a new system in which the gene encoding mScarlet was replaced by a kanamycin resistance gene (i.e., encoding aminoglycoside 3′-phosphotransferase). In a previous work, a system based on the light-inducible Cre recombinase was engineered to control the expression of a series of antibiotic resistance genes.^29^ Of note, our system presents the advantage of reversibility and a faster response ability. Using a concentration gradient of kanamycin, we determined the dose–response curves of the sRNA-modulated system according to different input signals (Figure 2A). Cells exposed to blue light exhibited resistance up to 5000 μg/mL, irrespective of aTc induction. However, under dark conditions, we found a shift of the response with aTc toward increased susceptibility. For instance, at 1000 μg/mL kanamycin in the dark, cells expressing the sRNA due to aTc induction showed substantially lower resistance than cells not induced (two-tailed Welch’s *t*-test, *P* = 2·10^–3^), in tune with a much lower basal production of aminoglycoside 3′-phosphotransferase as a result of riboregulation. Next, we carried out a study of cell viability after antibiotic treatment in which colony forming units (CFUs) were measured following dilution, showing agreement with absorbance quantifications (Figure 2B). In addition, we cultured cells on solid media with antibiotic, finding the formation of a lawn only upon exposure to blue light (Figure 2C). These results may pave the way for implementing complex spatiotemporal control strategies of cell survival applied to the management and containment of mixed bacterial populations.

**Figure 2:**
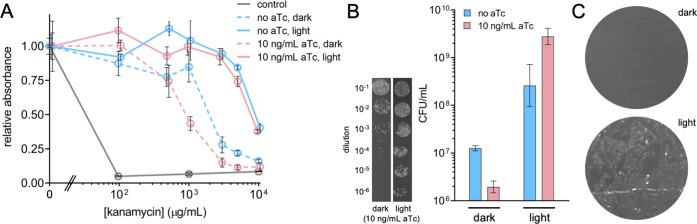
Light-activated kanamycin resistance with a digitized Opto-T7 system. A) Dose–response curves for different input conditions (light/dark, with/without aTc). Absorbance measured at 600 nm. B) CFU counts from different cultures at 1000 μg/mL kanamycin. Inset on the left, images of spot-plated cultures. C) Images of plates containing 3000 μg/mL kanamycin seeded with bacteria grown with blue light or in the dark. System implemented with pMagFast1 and nMagHigh1. Represented data in A,B) correspond to means ± standard deviations (*n* = 4).

In summary, we have significantly increased the dynamic range of the Opto-T7 optogenetic system by incorporating a *tet*-controlled riboregulatory module that allows minimizing the reconstitution of T7Pol under dark conditions. This module introduces an additional layer of post-transcriptional regulation and leverages the photosensitivity of aTc, ensuring minimal background activity while maintaining robust induction upon blue light exposure. This refinement aligns with ongoing efforts to enhance the performance of optogenetic tools for biotechnological and biomedical applications. In the case of bioproduction, for example, a tight control of gene expression is required for working with toxic proteins or enzymes that create severe metabolic burden.^30^ In cell-based therapeutic applications, digitized systems are crucial for reducing inflammation in the host organism in absence of input signal and to avoid nonspecific actions that may lead to concerning outcomes in terms of safety and containment.^31^ Furthermore, *tet*-controlled riboregulatory modules might be used to enhance the dynamic range of alternative optogenetic systems in bacteria, such as the heterologous two-component system controlling LacI (OptoLAC)^10^ or the AraC DNA-binding domain coupled to Vivid (BLADE).^32^ All in all, as our ability to engineer and refine synthetic mechanisms for the conditional activation or repression of gene expression advances, exploiting a diverse number of input signals, including light, greater possibilities arise for designing complex gene circuits from which to (re)­program living cells.

## Materials and Methods

### Strains, Plasmids, and Reagents

*E. coli* DH5αZ1 cells (*lacI* ^+^, *tetR* ^+^)^33^ were used to construct the plasmids following standard procedures. *E. coli* DH10B-ALT cells (*araC* ^+^, *lacI* ^+^, *tetR* ^+^)^34^ were used to express the genetic circuits for functional characterization. This strain was cotransformed with two plasmids, one containing the Opto-T7 system (pSC101 ori, CmR; see maps in Figure S7) and another containing the reporter gene (pBR322 ori, AmpR; see maps in Figure S8). Different versions of the Opto-T7 system were considered in which Magnets varied, implemented in the pAB203, pAB202, and pAB152 plasmids (acquired from Addgene; refs #101675, #101674, and #101663, respectively).^8^ Two different reporter plasmids were constructed. First, the pOPTO03 plasmid was obtained by subcloning the gene coding for mScarlet^35^ to be under the control of a T7 promoter. Then, the pOPTO12 plasmid was constructed by introducing the *tet*-controlled riboregulatory module (chemically synthesized by IDT) into pOPTO03. A P_Ltet_ promoter with different operators^36^ was used to enhance stability. To carry out antibiotic resistance assays, an additional plasmid was constructed by replacing the *mScarlet* gene with a KanR gene (pOPTO15). Empty plasmids derived from pAB203 and pOPTO03 were also constructed by removing the corresponding coding sequences. Luria–Bertani (LB, originally standing for lysogeny broth) medium was used for overnight preculturing and M9 minimal medium (1× M9 minimal salts, 2 mM MgSO_4_, 0.1 mM CaCl_2_, 0.05% thiamine, 0.05% casamino acids, and 0.4% glucose) for circuit characterization. The M9 medium was supplemented with arabinose and aTc when appropriate. Arabinose was used to induce the Opto-T7 system at different concentrations of up to 10 mM. aTc was used to induce the sRNA expression at different concentrations of up to 100 ng/mL. Typically, arabinose was used at 10 mM and aTc at 10 ng/mL. LB-agar solid medium was also used in the resistance assays. Ampicillin and chloramphenicol were the antibiotics used for plasmid selection at the concentrations of 50 and 34 μg/mL, respectively. Kanamycin was used for light-controlled resistance assays at different concentrations up to 10000 μg/mL. Compounds were obtained from Merck.

### Fluorescence Quantification

Precultures (2 mL) inoculated from single colonies of transformed *E. coli* DH10B-ALT cells (three replicates) were grown overnight in LB medium with shaking (220 rpm) at 37 °C. They were diluted 1:100 in 200 μL of fresh M9 medium supplemented with the appropriate inducers in a microplate (96 wells, black, clear bottom; Corning). These cultures were incubated with shaking (350 rpm) at 37 °C in a suitable platform (Innova 42R, Eppendorf) for light irradiation. For the cell preincubation with the chemical inducers, overnight precultures were diluted 1:100 in fresh M9 medium with 10 mM arabinose and 10 ng/mL aTc and were incubated with shaking at 37 °C for 5 h. Then, they were diluted 1:50 in 200 μL fresh M9 medium supplemented with the appropriate inducers in a microplate and were incubated in the platform for light irradiation. For the induced reversibility assay, overnight precultures were diluted 1:100 in fresh M9 medium with 1 mM arabinose and 10 ng/mL aTc and were incubated with shaking at 37 °C for 3 h. Then, they were diluted 1:200 in 200 μL fresh M9 medium supplemented with the same inducers in a microplate and were incubated in the platform under dark conditions. Blue light-emitting diodes (LEDs; HEGEHE) were placed 10 cm above the microplate. These LEDs produced a blue light of ∼470 nm with an intensity of >1 W/m^2^. For the incubations under dark conditions, the microplate was wrapped in aluminum foil. At different times (up to 6 h), the microplate was assayed in a multimode plate reader (CLARIOstar Plus, BMG) to measure absorbance (600 nm) and red fluorescence (excitation: 570 nm, emission: 610 nm). Mean background values of absorbance and red fluorescence, corresponding to the M9 medium, were subtracted to correct the signals. Normalized red fluorescence values were calculated as the ratio between the corrected red fluorescence and absorbance values in exponential phase of bacterial growth (OD_600_ ≈ 0.6). The mean value of normalized red fluorescence corresponding to transformed cells with empty plasmids was subtracted to obtain a final estimate of mScarlet intracellular production.

### Microscopy Imaging

Overnight precultures of transformed *E. coli* DH10B-ALT cells (two replicates) were diluted 1:100 in 200 μL of fresh M9 medium supplemented with the appropriate inducers in a microplate (96 wells, black, clear bottom; Corning). These cultures were incubated with shaking (350 rpm) at 37 °C in the Innova 42R platform (Eppendorf) under dark conditions. At an OD_600_ ≈ 0.6, samples were collected and visualized in an inverted fluorescence microscope (THUNDER, Leica) using yellow light irradiation (575 nm, 20% intensity, 400 ms exposure time) and a 100× objective. The commercial software provided by Leica was used to adjust the visualization of differential fluorescence among samples.

### Antibiotic Resistance Assay

Overnight precultures of transformed *E. coli* DH10B-ALT cells (four replicates) were diluted 1:200 in 200 μL of fresh M9 medium supplemented with the appropriate inducers in a microplate (96 wells, black, clear bottom; Corning). These cultures were incubated with shaking (350 rpm) at 37 °C in the Innova 42R platform (Eppendorf) for light irradiation. After 2 h, kanamycin was added at different concentrations. For the incubations under dark conditions, the microplate was wrapped in aluminum foil. At different times (up to 8 h), the microplate was assayed in a multimode plate reader (CLARIOstar Plus, BMG) to measure absorbance (600 nm). Mean background value of absorbance, corresponding to M9 medium, was subtracted to correct the signals. As a negative control, transformed cells with empty plasmids were used. For comparative purposes, final absorbance values for each kanamycin concentration were normalized by the final absorbance of the culture grown without antibiotic (OD_600_ ≈ 1). In addition, CFUs were measured by following a microspotting protocol in the dark. Cultures grown with and without aTc and with 1000 μg/mL kanamycin were serially diluted up to 1:10^9^ in fresh M9 medium in a microplate. Cultures (10 μL) were then spot-plated on LB-agar. Plates were incubated overnight at 37 °C. The mean number of colonies per condition was recorded. To validate antibiotic resistance in solid medium, overnight precultures (10 μL) were plated on LB-agar supplemented with 10 mM arabinose, 10 ng/mL aTc, and 3000 μg/mL kanamycin. Plates were incubated overnight at 37 °C with blue light or in the dark.

## Supplementary Material



## Notes

Sequence schematics of the *tet*-controlled riboregulatory module (Figure S1), additional experimental results of light-controlled expression (Figures S2–S6), and plasmid maps (Figures S7, S8) (PDF)
