# Supplementary material for: Digitizing the Blue Light-Activated T7 RNA Polymerase System with a tet-Controlled Riboregulator
Source: ACS Synth Biol. 2025 May 19;14(6):2393–9. doi: 10.1021/acssynbio.5c00142 (PMC12186670; doi:10.1021/acssynbio.5c00142)
Supplement: Supplementary file 1 [file sb5c00142_si_001.pdf]

Supplementary Material

Digitizing the blue light-activated split T7 RNA polymerase system with a *tet*-controlled riboregulator

Sara Baldanta[1] and Guillermo Rodrigo[1,*]

[1]Institute for Integrative Systems Biology (I2SysBio), CSIC – University of Valencia, 46980 Paterna, Spain

[*]Correspondence: guillermo.rodrigo@csic.es

P_{Ltet} promoter:

-35 -10

ACTCTATCATTGATAGAGTTTGACATCCCTATCAGTGATAGAGATACTGAGCAC

*tetO*1 *tetO*2

T7Pol-targeting sRNA gene:

▼cleavage site

*GAACACCTGATGAGTCCGTGAGGACGAAACGAGCTAGCTCGTC****GTGTTC*****ATAGATCTTTA**
CCTATTTGTAGAAATATTTTATTCGCCCCCGGAAGATCATTCCGGGGGCTTTTTTATT

italic: hammerhead ribozyme
bold: antisense RNA
red: terminator (Hfq-recruiting element)

T7Pol(N)-nmag gene:

targeted region

TTGGAAGGAGGTAAAGATCTATGAACACGATTAACATCGCTAA...

Shine Dalgarno — start codon

Figure S1. Sequence schematics of the different elements involved in the sRNA-based regulation. The P_{Ltet} promoter contained two different operators (*tetO*$_1$ and *tetO*$_2$).

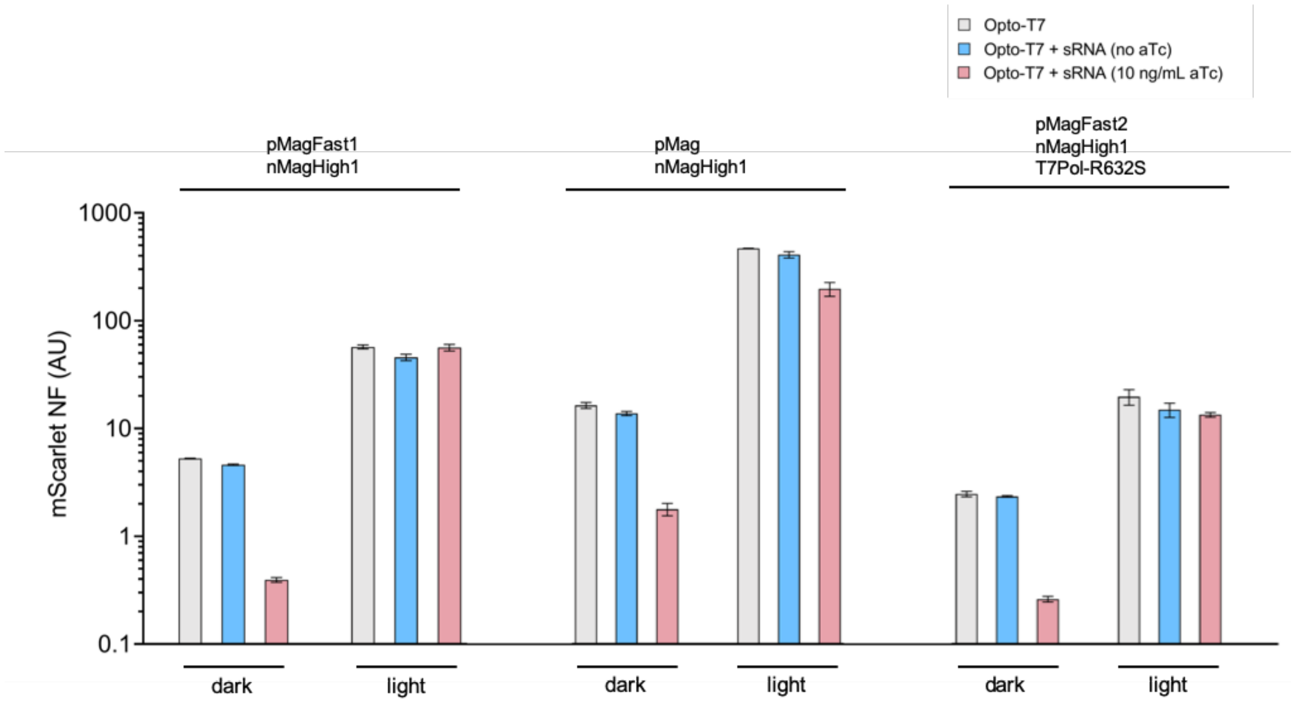

Figure S2. Fluorescence-based reporter gene expression analyses (light/dark, with/without aTc) using the same fluorescence scale for a comparative assessment between different implementations. Represented data correspond to means ± standard deviations (*n* = 3).

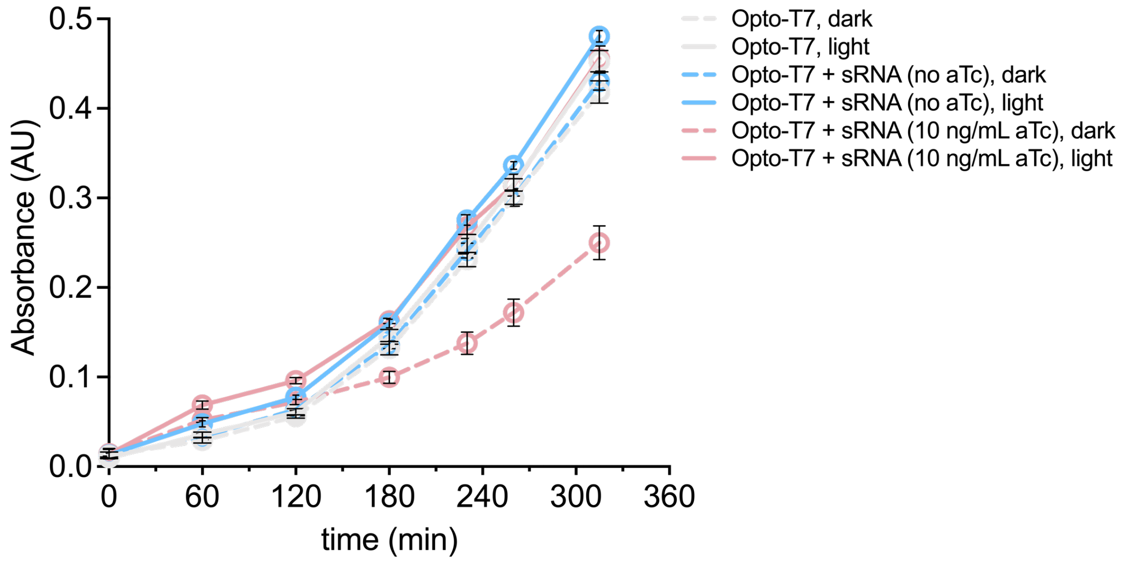

Figure S3. Cell growth curves for different induction conditions with aTc and blue light. Represented data in the plot correspond to means ± standard deviations (*n* = 3). Absorbance measured at 600 nm.

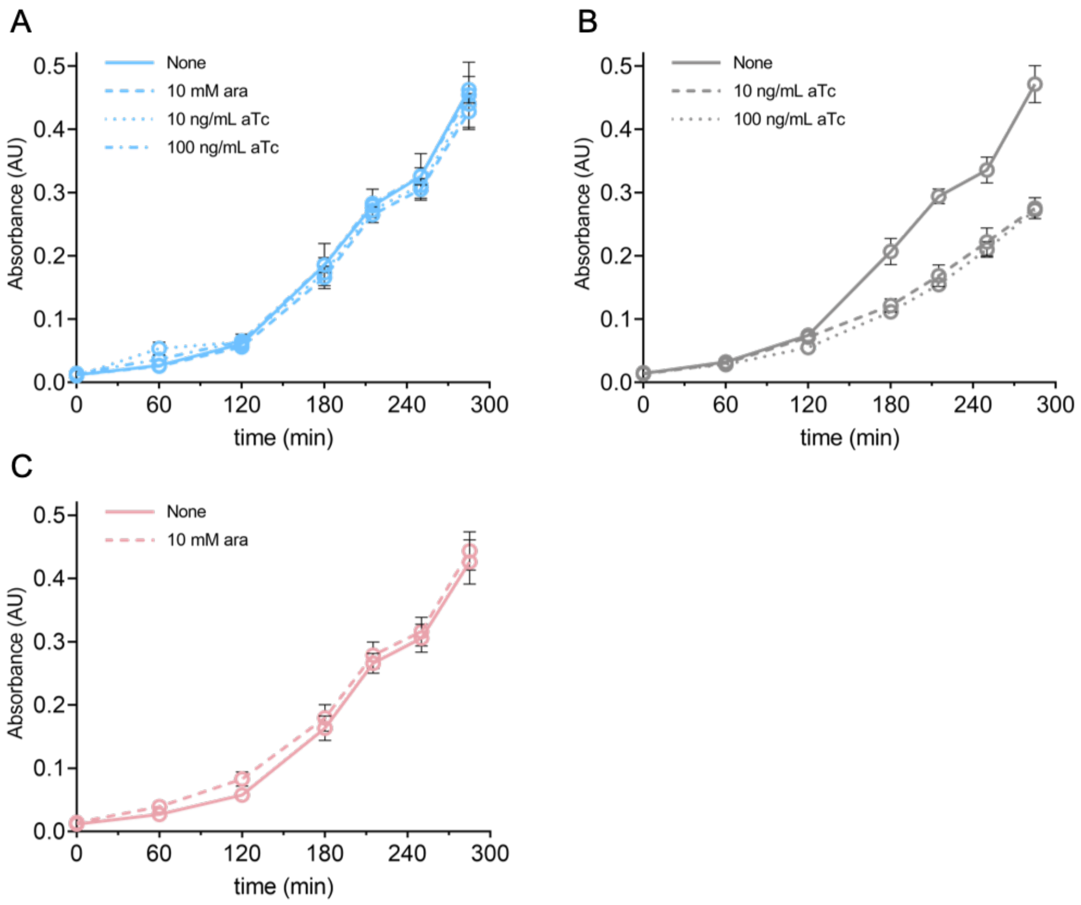

Figure S4. Control cell growth curves for different chemical induction conditions in the dark. A) Plain cells. B) Cells transformed with pOPTO12. C) Cells transformed with pAB203. Represented data in the plot correspond to means ± standard deviations (n = 3). Absorbance measured at 600 nm.

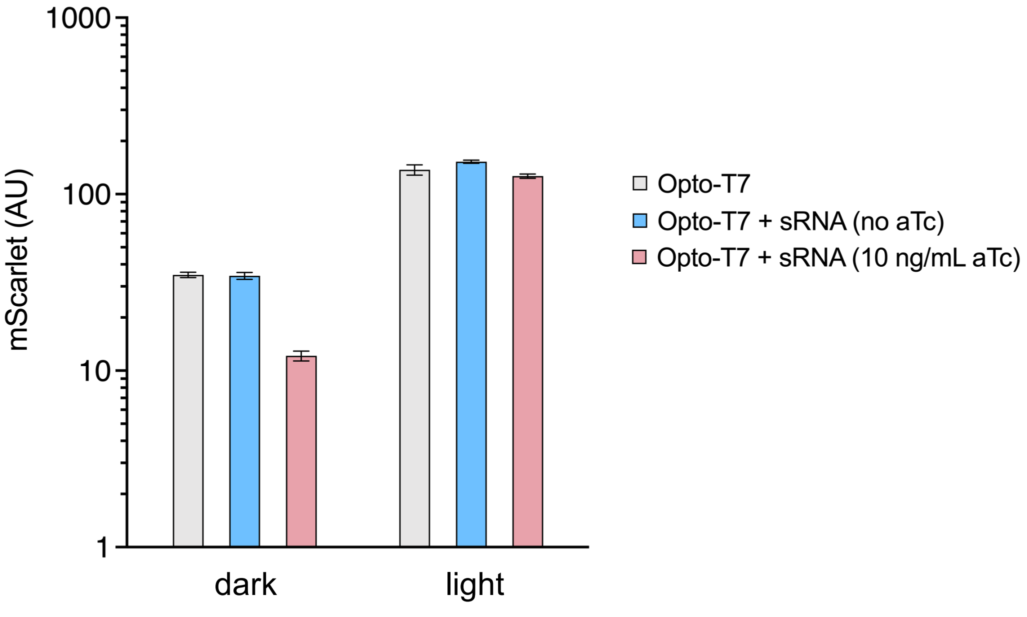

Figure S5. Fluorescence-based reporter gene expression analysis at 28 ºC. System implemented with pMagFast1 and nMagHigh1. Represented data in the bar plot correspond to means ± standard deviations (n = 3).

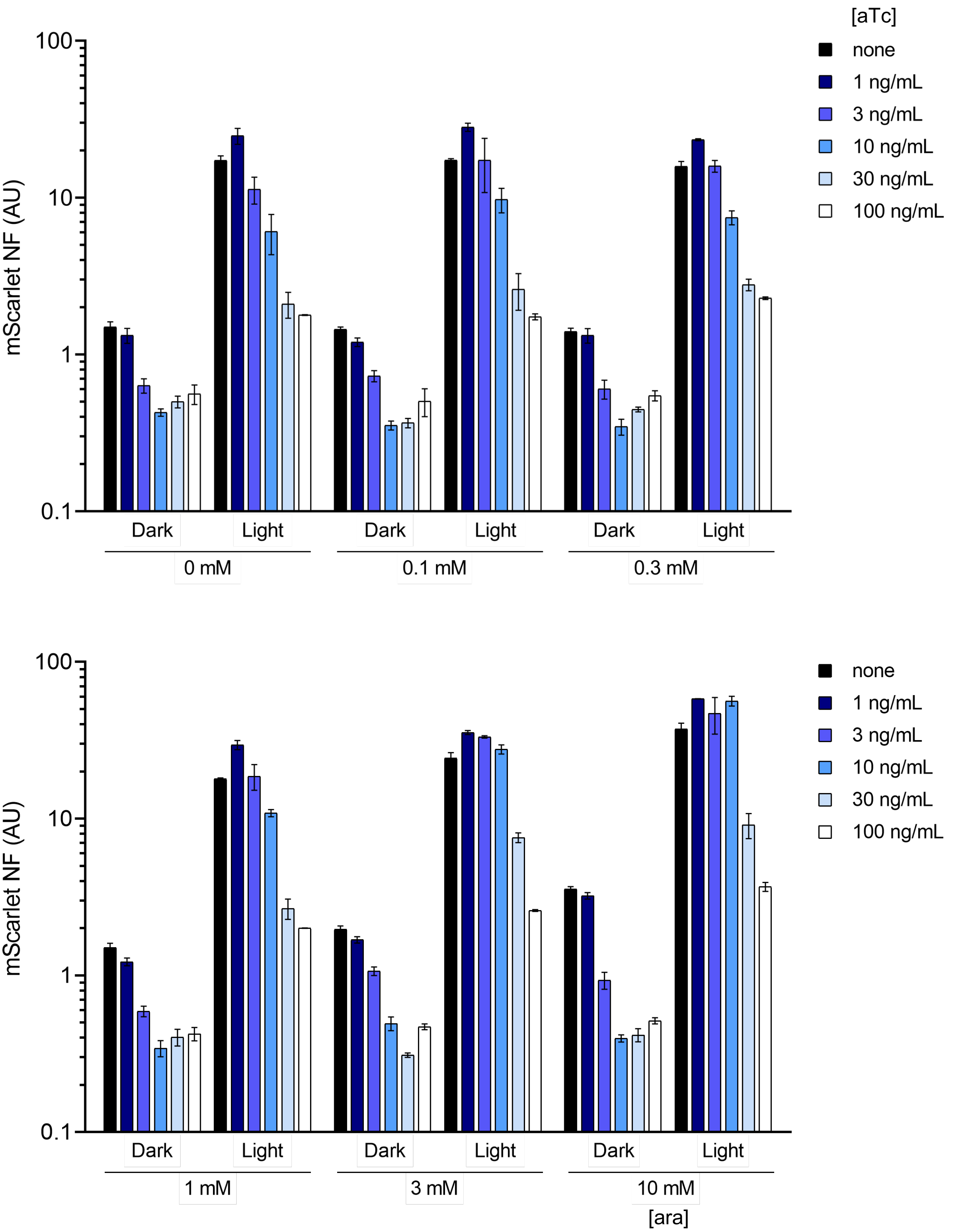

Figure S6. Fluorescence-based reporter gene expression analysis for a double concentration gradient of arabinose and aTc, leading to 36 combinations (72 input conditions considering light). System implemented with pMagFast1 and nMagHigh1. Represented data in the bar plot correspond to means ± standard deviations (n = 3).

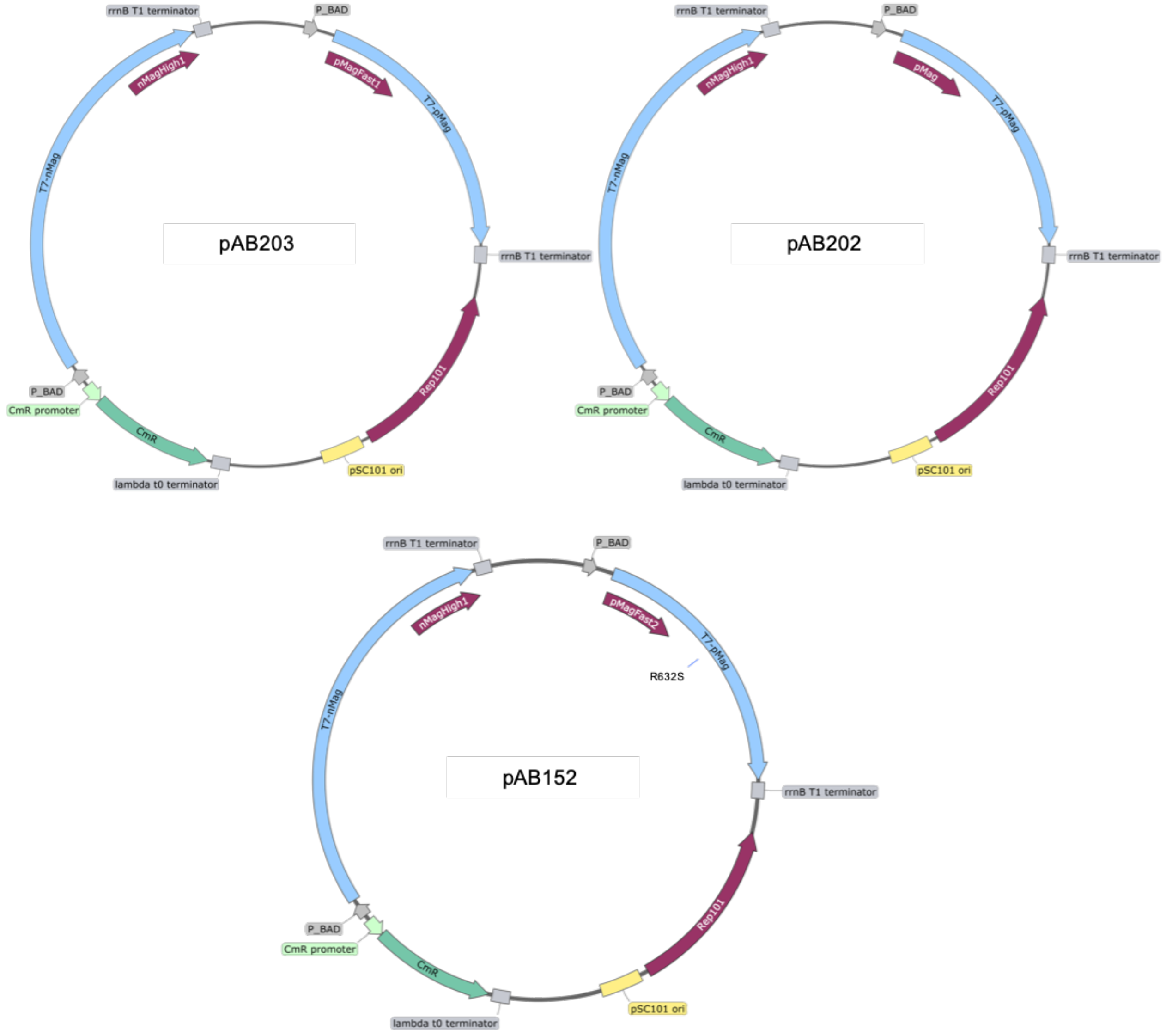

Figure S7. Maps of the Opto-T7 plasmids used to deploy the synthetic gene circuits. pAB203, pAB202, and pAB152 express different Opto-T7 systems implemented with different Magnets versions.

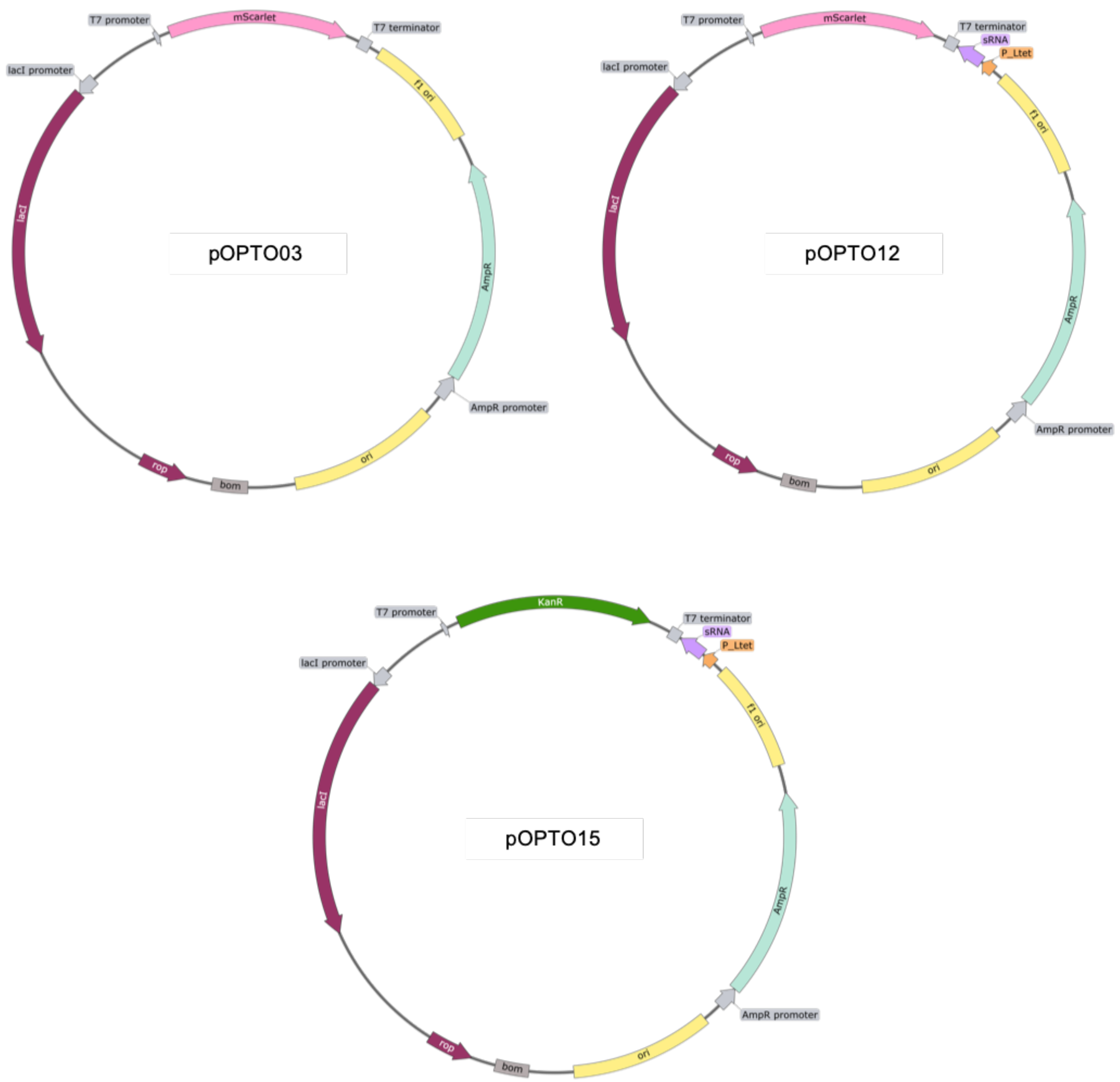

Figure S8. Maps of the reporter plasmids used to deploy the synthetic gene circuits. pOPTO03 only expresses the reporter (mScarlet), pOPTO12 expresses the reporter and the *T7Pol*-targeting sRNA, and pOPTO15 expresses a kanR gene together with the sRNA.
